# Particulate matter_10_-induced airway inflammation and fibrosis can be regulated by chitinase-1 suppression

**DOI:** 10.1186/s12931-023-02392-8

**Published:** 2023-03-18

**Authors:** Yong Jun Choi, Heejae Han, Jae-Hyun Lee, Jaeuk Lee, Chi Young Kim, Min Kwang Byun, Jae Hwa Cho, Hye Jung Park

**Affiliations:** Division of Pulmonology, Department of Internal Medicine, Yonsei University College of Medicine, Gangnam Severance Hospital, 211, Eonju-Ro, Gangnam-Gu, Seoul, 06273 Korea

**Keywords:** Chitinase, Particulate matter, RNA sequencing, Lung

## Abstract

**Background:**

Particulate matter_10_ (PM_10_) can induce airway inflammation and fibrosis. Recently, chitinase-1 has been shown to play key roles in inflammation and fibrosis. We aimed to investigate the effects of chitinase-1 inhibitor in PM_10_-treated murine mice models.

**Methods:**

In female BALB/c mice, PM_10_ was intranasally administered six times over 3 weeks, and ovalbumin (OVA) was intraperitoneally injected and then intranasally administered. Chitinase-1 inhibitor (CPX) 6 times over 3 weeks or dexamethasone 3 times in the last week were intraperitoneally administered. Two days after the last challenges, mice were euthanized. Messenger RNA sequencing using lung homogenates was conducted to evaluate signaling pathways.

**Results:**

PM_10_ and/or OVA-induced airway inflammation and fibrosis murine models were established. CPX and dexamethasone ameliorated PM_10_ or PM_10_/OVA-induced airway hyper-responsiveness, airway inflammation, and fibrosis. CPX and dexamethasone also reduced levels of various inflammatory markers in lung homogenates. PM_10_ and OVA also induced changes in mRNA expression across an extreme range of genes. CPX and dexamethasone decreased levels of mRNA expression especially associated with inflammation and immune regulation. They also significantly regulated asthma and asthma-related pathways, including the JACK-STAT signaling pathway.

**Conclusions:**

Chitinase-1 suppression by CPX can regulate PM_10_- and OVA-induced and aggravated airway inflammation and fibrosis via an asthma-related signaling pathway.

**Supplementary Information:**

The online version contains supplementary material available at 10.1186/s12931-023-02392-8.

## Introduction

As one of the major components of air pollution, particulate matter_10_ (PM_10,_ airbone particles < 10 μm)—which includes various heavy metals such as copper, lead, silicon, and aluminum—has recently become an important issue for population health outcomes. For example, it was the seventh largest risk factor for disability-adjusted-life years between 2000 and 2015 [1–3]. PM_10_ also causes harmful health effects in various diseases, including cardiovascular disease, respiratory disease, and lung cancer and mortality [4]. PM_10_ enters the airway through inhalation and can cause injury to the respiratory tract [5]. Previous epidemiologic and experimental studies have demonstrated that harmful effects of PM_10_ can be prominent in underlying respiratory diseases, including asthma and chronic obstructive pulmonary disease (COPD) [6, 7]. We also showed previously that inhalation of PM_10_ induces airway inflammation and fibrosis with changing RNA expression associated with inflammation and immune response [8]. However, therapeutic options in PM_10_-induced airway diseases have not been well studied.

Chitinase-1 is one of the main chitinases that can degrade chitin [9]. Chitinase-1 is expressed in alveolar macrophages and neutrophils and has a critical role in the innate immune response of the respiratory tract [10, 11]. Exposure to cigarette smoke and environmental toxins can enhance production and activity of chitinase-1, and extensive increase of chitinase-1 levels leads to airway inflammation and fibrosis [12]. Thus, chitinase-1 inhibitor targeting chitinase-1 has been studied as a therapeutic options for airway inflammation and fibrosis in recent experimental airway-diseases models [13, 14]. The effects of chitinase-1 inhibitor were activated via TGF-b signaling [15]. Thus, we hypothesized that chitinase-1 inhibitor can improve PM_10_-related respiratory disease by improving inflammation and fibrosis, similar to previous studies using different disease models [15].

In this study, we aimed to investigate the ability of chitinase-1 inhibitor (CPX) to ameliorate airway inflammation and fibrosis in PM_10_-treated murine models.

## Materials and methods

### Animal model designs

Female BALB/c mice, between 5 and 6 weeks old (Orient, Daejeon, Korea), were maintained at conventional animal facilities under pathogen-free conditions, and 5 mice were assigned to each group. To establish the PM_10_-induced murine model (PM_10_ model), PM_10_ (ERMCZ-120^®^ certified reference material; Sigma-Aldrich, St Louis, MO, USA; 100 μg suspended in 20 μL normal saline was intranasally administered 6 times over 3 weeks. The dose of PM_10_ was chosen based on a previous study [8]. OVA is a well-known allergen used to induce asthma in a mouse model.^2^

**Fig. 1:**
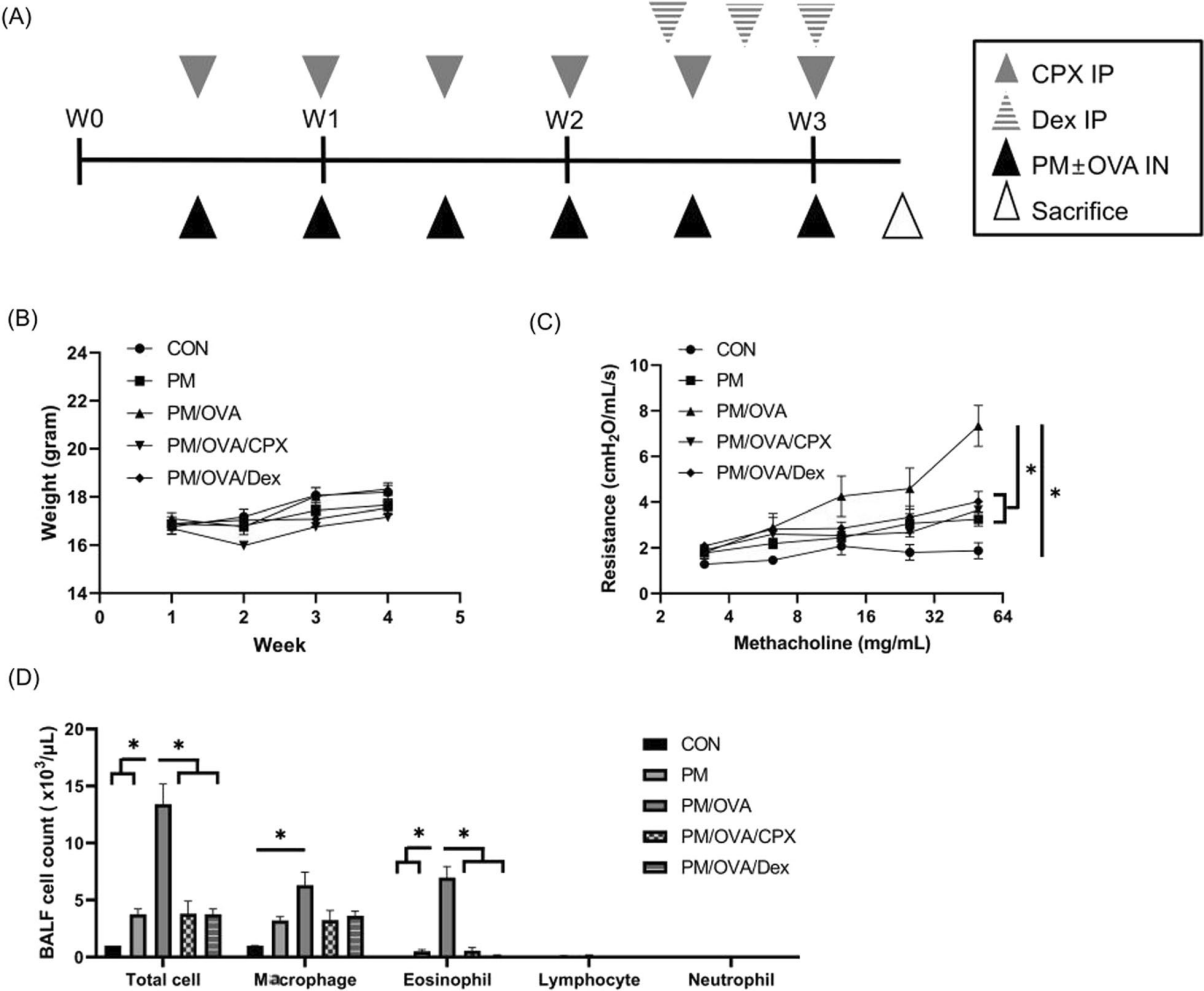
Study scheme (**A**), weight change (**B**), airway hyper-responsiveness (**C**), and bronchoalveolar lavage fluid analysis (**D**). *CPX* chitinase-1 inhibitor, *Dex* dexamethasone, *IP* intraperitoneally injection, *IN* intranasally administration, *OVA* ovalbumin, *BALF* bronchoalveolar lavage fluid. Data are presented as mean ± standard deviation. *P < 0.05 between them

Dexamethasone was used as positive control since it has strong anti-inflammatory effect [16]. To establish the chronic OVA-induced asthma murine model, mice were challenged intranasally with 30 µL of OVA (1 mg/mL) (Sigma-Aldrich, St Louis, MO, USA) in saline solution 6 times over 3 weeks. An OVA/PM_10_-treated model was established with the 6 concurrent treatments mentioned above (PM_10_ and OVA) over 3 weeks. Chitinase-1 inhibitor (CPX, Sigma-Aldrich, St Louis, MO, USA, 100 mg/kg, intraperitoneally) was administered 6 times over 3 weeks and treated with dexamethasone (Sigma-Aldrich, St Louis, MO, USA, 3 mg/kg, intraperitoneally) 3 times in the final week. Dexamethasone, which has a strong anti-inflammatory effect, was used as positive control. Mice body weights were measured weekly. All mice were euthanized 2 days after their last treatment (Fig. 1A).

We divided mice into 5 groups: control (only treated with saline), PM (PM_10_-treated model), PM/OVA (PM_10_ and OVA-treated model to maximize airway and lung inflammation), PM/OVA/CPX (PM_10_, OVA, and CPX-treated model to reveal effects of CPX on inflammation), and PM/OVA/Dex (PM_10_, OVA, and Dex-treated model to confirm effects of Dex as a positive control).

All experimental procedures of mice studies were approved by the Institutional Animal Care and Use Committee, Animal Research Ethics Board of Yonsei University (Seoul, Korea) (IACUC approval number, 2020-0087) and were performed in accordance with the Committee’s guidelines and regulations for animal care.

### Measurement of airway hyper-responsiveness

Airway hyper-responsiveness (AHR) to inhaled aerosolized methacholine (MCh; Sigma-Aldrich, St Louis, MO, USA) was measured using a forced oscillation technique (FlexiVent; SCIREQ, Montreal, QC, Canada) on the euthanasia day, as described in a previous study [17–19]. Aerosolized phosphate-buffered saline (PBS) or methacholine at varying concentrations (3.125 mg/mL, 6.25 mg/mL, 12.5 mg/mL, 25.0 mg/mL, or 50.0 mg/mL) was administered to mice for 10 s via a nebulizer connected to a ventilator. Then, AHR was assessed by measurements of airway resistance.

### Inflammatory cell counting in bronchoalveolar lavage fluid

To collect bronchoalveolar lavage fluid (BALF), lung lavage was performed, using 1 mL of Hank’s balanced salt solution (HBSS) through a tracheal tube. The recovered BALF was centrifuged and resuspended in 300 µL HBSS. Total cell numbers were determined using a hemocytometer and trypan blue staining. BALF cells were centrifuged by cytocentrifugation (Cytospin 3; ThermoFisher Scientific, Waltham, MA, USA) and were pelleted to cytospin slides. The slides were stained with hematoxylin and eosin (H&E Hemacolor^®^, Merck, Darmstadt, Germany), and a differential count of inflammatory cells was performed (200 cells per slide).

### Histological analysis

The remnant lung, i.e., the lung that was not used for BALF collection, was fixed in 4% formalin and embedded in paraffin. Lung sections were cut into 3–4-µm-thick slices and stained with H&E, periodic acid-Schiff (PAS), and Masson trichrome (M&T) for histological analysis. The slides were observed under a light microscope (× 200 magnification). The area of fibrosis was measured by estimating the color-pixel count over the pre-set threshold color on MT-stained slides at 200 × magnification using MetaMorph program (Molecular Devices, Sunnyvale, CA, USA).

### Lung homogenate

After collecting BALF, remaining lung tissue was resected and homogenized using a tissue homogenizer (Biospec Products, Bartlesville, OK, USA) in lysis buffer and protease inhibitor solution (Sigma-Aldrich, St Louis, MO, USA). After incubation and centrifugation, supernatants were harvested and passed through a 0.45-micron filter (Gelman Science, Ann Arbor, MI, USA). The final preparations were stored at − 20℃ for cytokine analysis as described previously [17].

### Analysis of cytokines

Concentrations of interleukin (IL)-1*β*, TNF-*α*, IL-6, IL-13, and TGF-*β* in lung homogenates were assessed via enzyme-linked immunosorbent assay (R&D Systems, San Diego, USA) according to the manufacturer’s instructions. All samples were assessed in duplicate.

### Immunofluorescence study

Lung tissue was fixed with 10% formalin for 24 h. Lung tissue sections were deparaffinized, permeabilized with 10 mM citrate buffer, and blocked with 5% bovine serum albumin. The slide was incubated with anti-chitinase-1 antibodies (1:80; Santacruz, TX, USA) overnight at 4 °C. Slides were washed five times in phosphate-buffered saline (PBS). After washing, the slides were incubated with m-IgGκ BP-conjugated FITC antibody (1:100; santacruz, CA, USA) and mounting medium with PI (Invitrogen, CA, USA) overnight at 4 °C. Images were acquired with Axio Imager M2 microscope (Carl Zeiss).

### Analysis of mRNA expression level

Total RNA was extracted from lung tissues using in TRIzol^®^ reagent (Ambion, Life technologies, Carlsbad, CA, USA) according to the manufacturer’s instructions. Reverse transcription was performed using reverse transcriptase (Invitrogen, Carlsbad, CA, USA) primed with oligo (dT) primer. The synthesized cDNAs were amplified using the SYBR^®^ green PCR master mix (BioRad, California, USA) and forward and reverse primers (Bioneer, Daejeon, Korea) using a real-time PCR system (StepOnePlus, Applied Biosystems, Foster City, CA, USA). The following primers were used for collagen III and collagen I, respectively: GTG AAA CTG GTG AAC GTG GC (F) and ATA GGA CCT GGA TGC CCA CT (R) for collagen III; GAG AGG TGA ACA AGG TCC CG (F) and AAA CCT CTC TCG CCT CCT GC (R) for collagen I.

### Library preparation and mRNA sequencing

Total RNA was extracted from lung tissue using Trizol reagent (Invitrogen). mRNA isolation was performed using the Poly(A) RNA Selection Kit V1.5 (LEXOGEN, Inc., Austria). The total RNA is briefly denatured and the polyadenylated 3' ends of mRNAs are hybridized for isolation. The isolated mRNAs were used for cDNA synthesis. Libraries were prepared using the NEBNext Ultra II Directional RNA Seq Kit (NEW ENGLAND BioLabs, Inc., UK). Indexing was performed using the Illumina indexes 1–12. The enrichment step was carried out using PCR. Subsequently, libraries were checked using the Agilent 2100 bioanalyzer (Agilent Technologies, Amstelveen, The Netherlands) to evaluate the mean fragment size. Quantification was performed using the library quantification kit with an ND 2000 Spectrophotometer (Thermo Inc., DE, USA) and StepOne Real Time PCR System (Life Technologies, Inc., USA). High-throughput sequencing was performed as paired end 100 sequencing using NovaSeq 6000 (Illumina, Inc., USA).

Quality control of raw sequencing data was performed using FastQC (Simon, 2010). Adapter and low-quality reads (< Q20) were removed using FASTX_Trimmer (Hannon Lab 2014) and BBMap (Bushnell 2014). Then, the trimmed reads were mapped to the reference genome using TopHat [20]. Gene expression levels were estimated by calculating fragments per kb per million reads (FPKM) using Cufflinks [21]. The FPKM values were normalized based on a quantile normalization method using EdgeR within R (R development Core Team 2016).

### Statistical analysis

All results are expressed as the mean ± standard error. The AHR data were analyzed using repeated-measure analysis of variance (ANOVA), followed by a post-hoc Bonferroni test. One-way ANOVA was performed to assess the significance of differences in BALF cell count, cytokine levels, and quantitative fibrosis among groups. All statistical analyses were performed with IBM SPSS version 18.0 (SPSS Inc., Chicago, IL, USA). *P*-values < 0.05 were considered statistically significant. All RNA sequencing analyses were performed by R (version 4.0.2; R Foundation for Statistical Computing, Vienna, Austria) software. Gene ontology (GO) and Kyoto Encyclopedia of Genes and Genomes (KEGG) enrichment analysis were conducted by “clusterProfiler” R package. Upset plot was plotted by “DOSE” R package, chord plot was plotted by “GOplot” R package and KEGG pathway was described by "pathview” R package.

## Results

### Effects of CPX on airway inflammation

The body weight of all mice increased over the course of the experiment, and the degree of weight changes was not significantly different among groups (Fig. 1B). The PM group did not induce significant AHR; however, the PM/OVA group showed significant AHR compared to the CON group. CPX or dexamethasone ameliorated AHR compared to the PM/OVA group (Fig. 1C). The PM/OVA group showed significant increase of total cell, macrophage, and eosinophil in BALF. CPX or dexamethasone significantly reduced levels of total cell and eosinophil in BALF (Fig. 1D).

### Effects of CPX on fibrosis

**Fig. 2:**
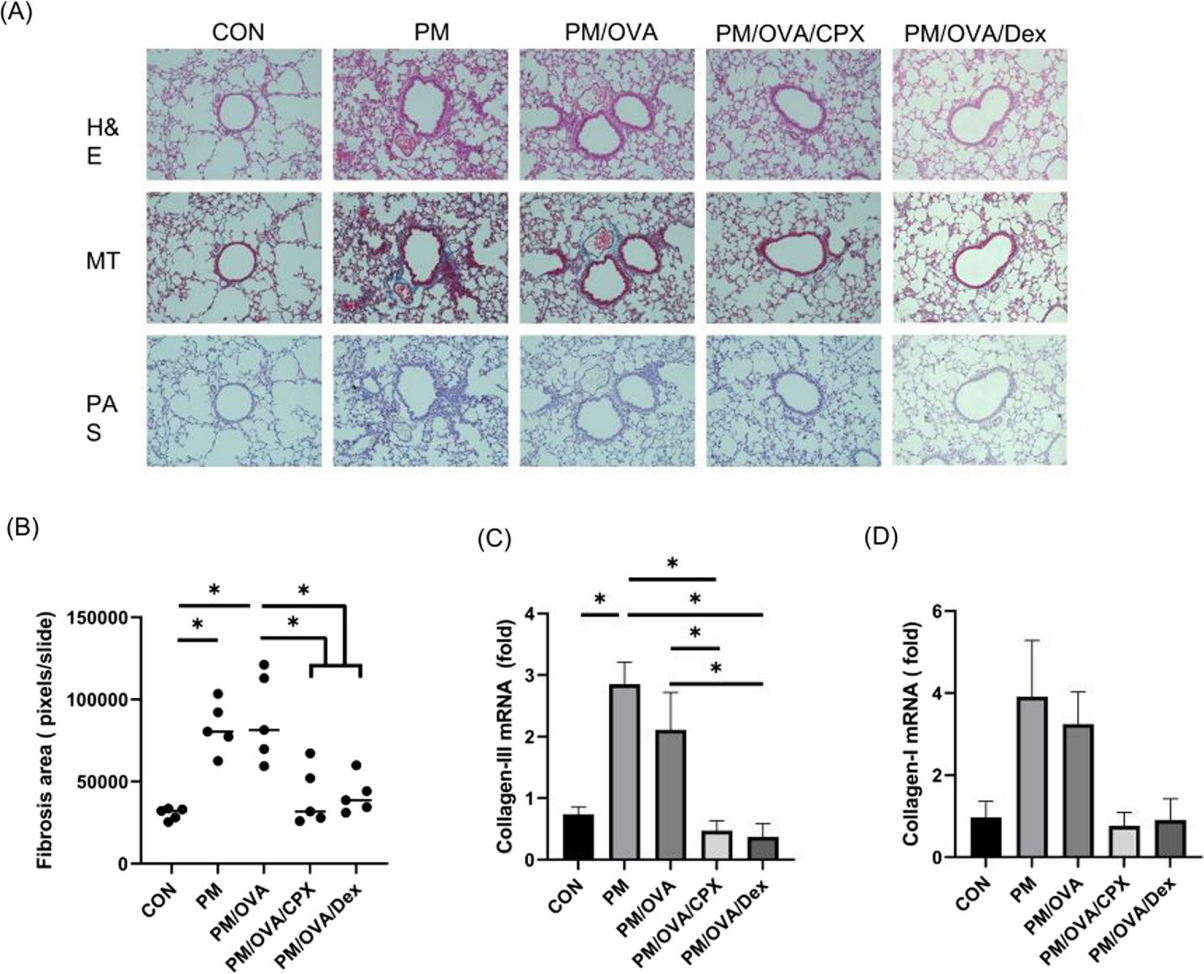
Pathologic findings (**A**) (H&E, PAS, and M&T; all × 200 magnification), quantitative fibrosis area (**B**), mRNA expression of collagen III (**C**), and mRNA expression of collagen-I (**D**). H&E, hematoxylin and eosin; PAS, periodic acid-Schiff; M&T, Masson trichrome. Data are presented as mean ± standard deviation. *P < 0.05 between them

The PM and PM/OVA groups showed significant fibrotic changes around the airway lumen and vessels in MT-staining pathologic findings. CPX or dexamethasone ameliorated these fibrotic changes (Fig. 2A). Quantitative analysis also showed that PM and OVA-induced significant increase of fibrotic changes, and CPX or dexamethasone improved them (Fig. 2B). The mRNA expression of collagen and fibrotic marker also increased in the PM and OVA-treated groups, and decreased in the CPX- or dexamethasone-treated group (Fig. 2C, D).

### Effects of CPX on inflammatory markers

**Fig. 3:**
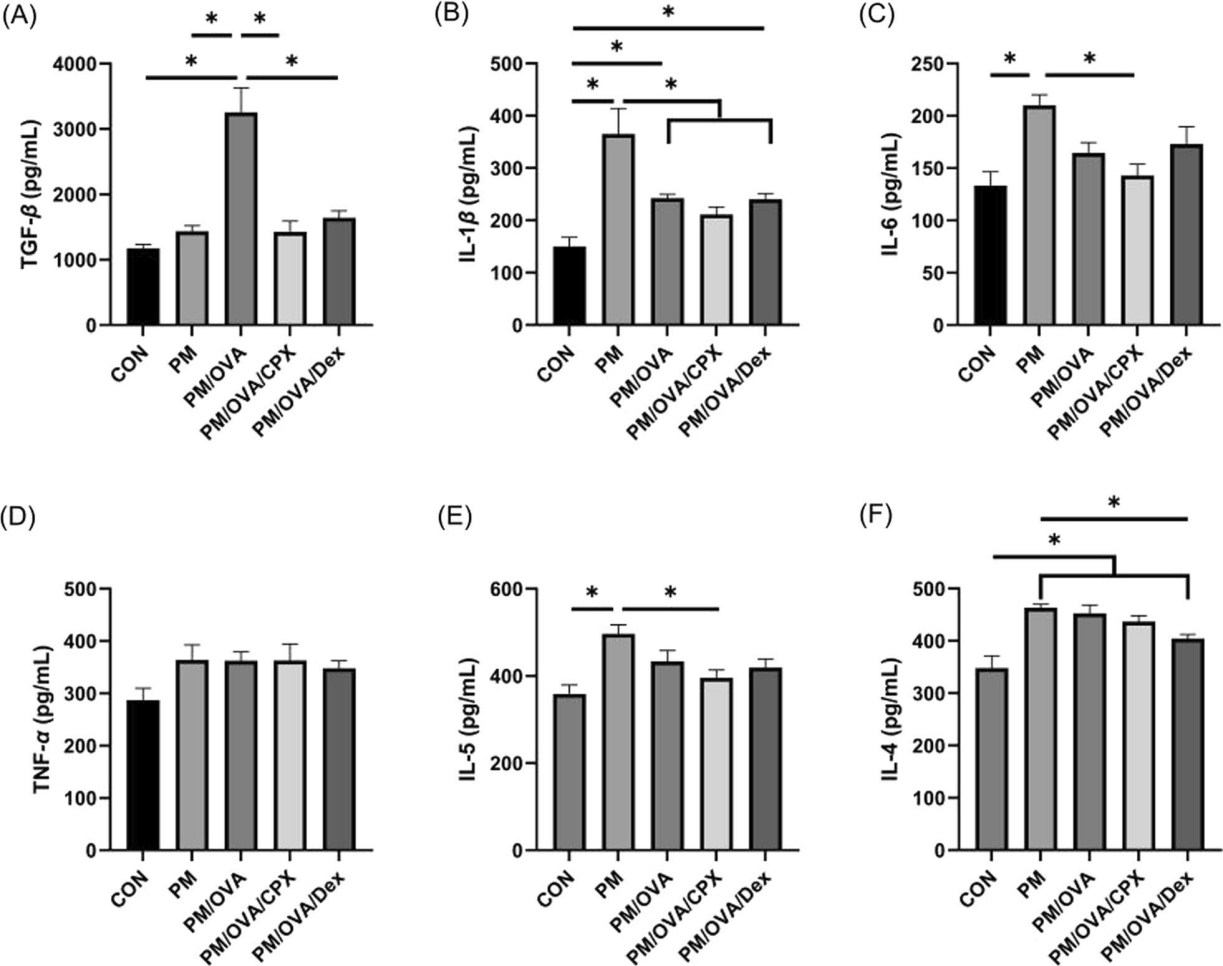
TGF-*β* (**A**), IL-1*β* (**B**), IL-6 (**C**), TNF-*α* (**D**), IL-5 (**E**), and IL-4 (**F**) levels in lung homogenates. Data are presented as mean ± standard deviation. *P < 0.05 between them

The level of TGF-*β* in lung homogenates were significantly increased in the PM/OVA group, and it was significantly decreased in the CPX-or dexamethasone-treated group (Fig. 3A). The level of IL-1*β* significantly increased in the PM group and PM/OVA group. That in CPX or dexamethasone treated group were decreased compared to that in PM group (Fig. 3B). The level of IL-6 was significantly increased in the PM group, but decreased in the CPX-treated group compared to the PM group (Fig. 3C). The levels of TNF-α were not significantly changed (Fig. 3D). The levels of IL-4 significantly increased in the PM and PM/OVA groups compared to those of the control group, and decreased in the PM/OVA/Dex group (Fig. 3E). The level of IL-5 in the PM group was significantly increased compared to that of the control group (Fig. 3F).

### CPX significantly suppressed target molecule, chitinase-1

**Fig. 4:**
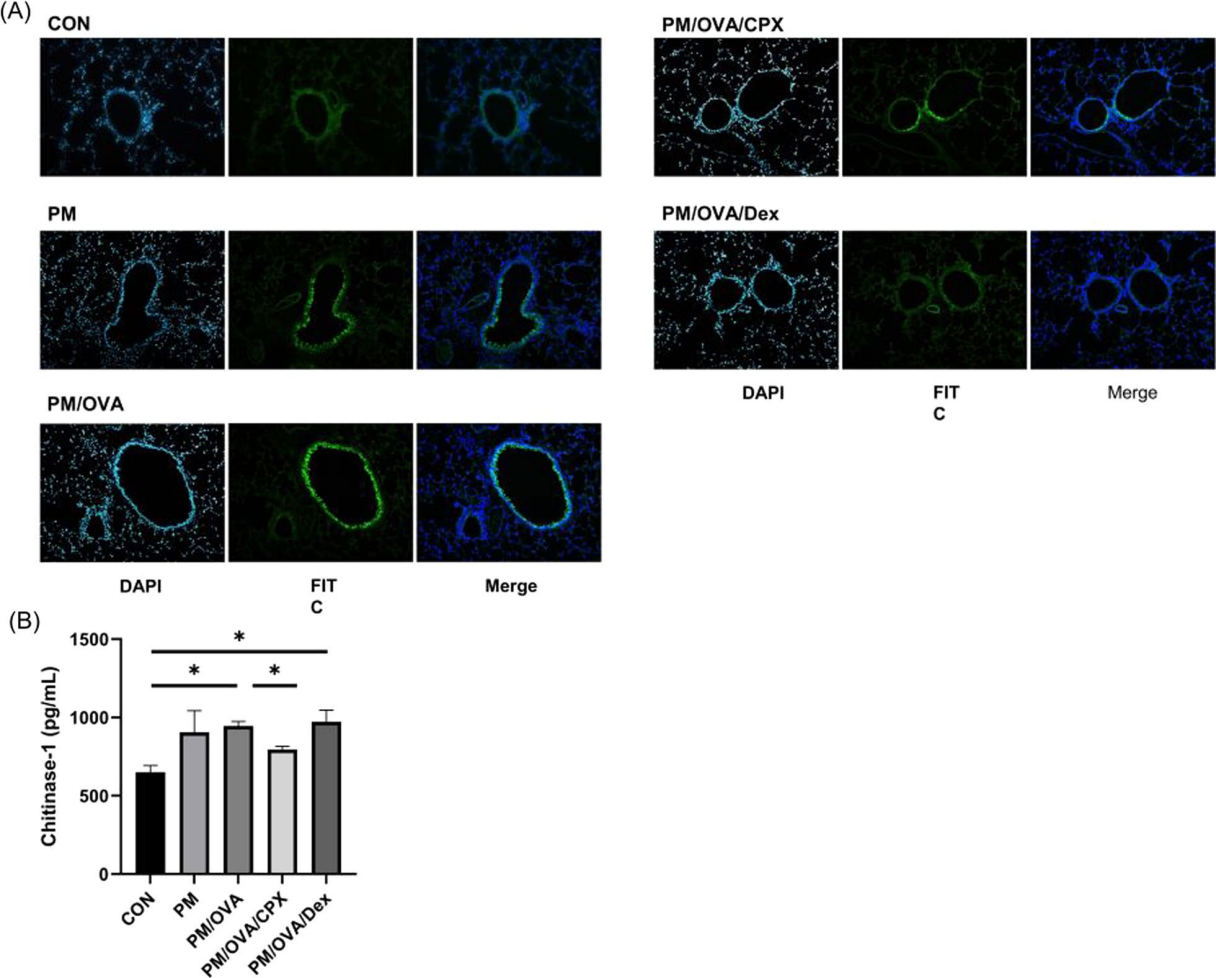
IF (**A**) and the levels of chitinase-1 in lung homogenates (**B**). *P < 0.05 between them

In the IF study, various cells, including bronchial epithelial cells and interstitial cells, expressed chitinase-1. The areas expressing chitinase-1 (green color on FITC and merge slides) were more obvious and larger in the PM and PM/OVA groups compared to that in the control group. They were ameliorated in PM/OVA/CPX and PM/OVA/Dex group (Fig. 4A). The level of chitinase-1 in lung homogenates were significantly increased in the PM and PM/OVA groups compared to that in the control group, and significantly decreased in the CPX- and dexamethasone-treated groups (Fig. 4B).

### Differential gene expression between groups

**Fig. 5:**
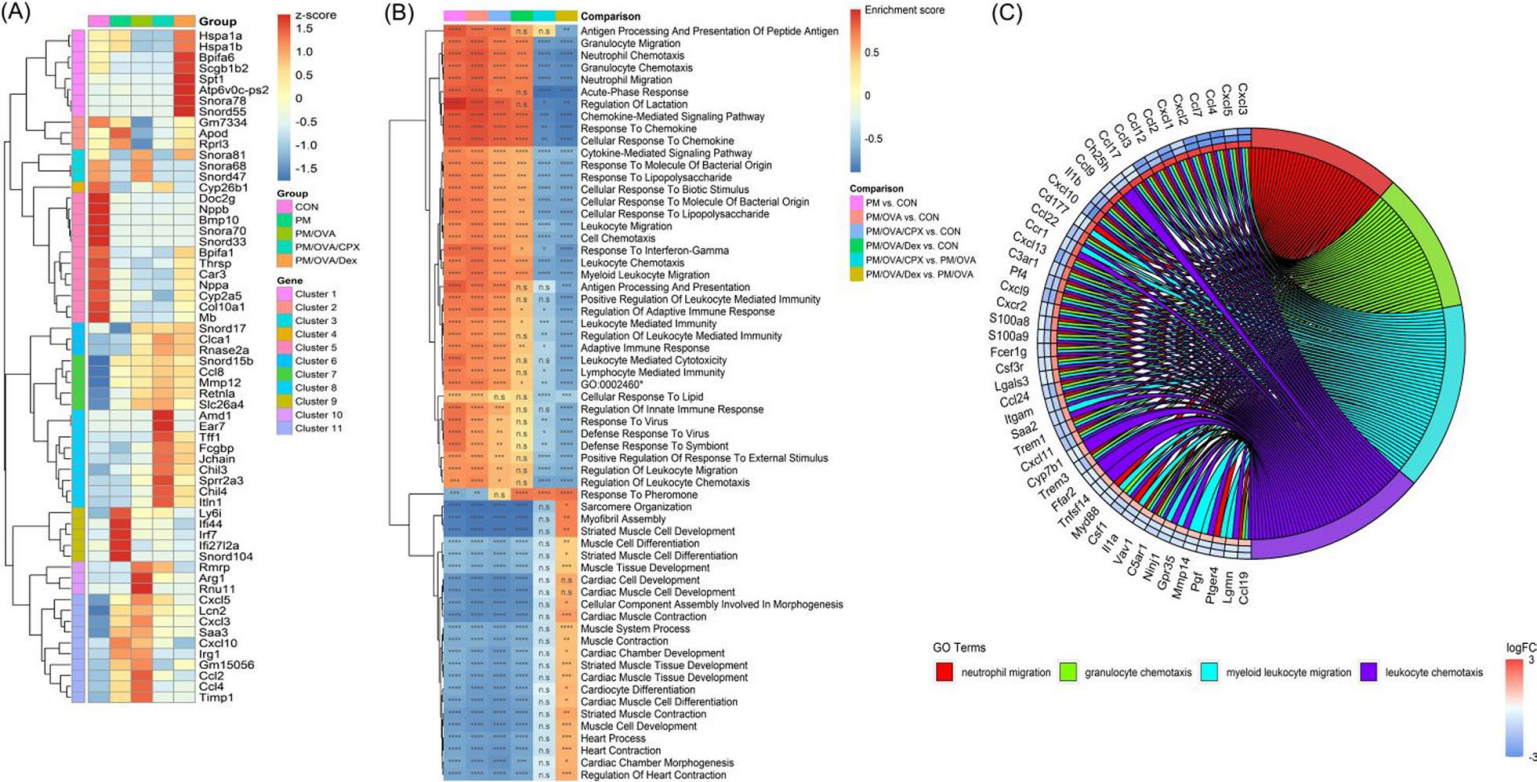
Differentially expressed genes and gene ontology analysis. Heatmap of top 20 highly variable gene expression of each groups (**A**), heatmap of top 20 gene ontology enrichment score in each groups (**B**), and chord diagram presenting differently expressed genes in commonly upregulated biologic process both PM/OVA/CPX and PM/OVA/Dex (**C**). GO: 0002460-adaptive immune response based on somatic recombination of immune receptors built from immunoglobulin superfamily domains. *adjusted *P* < 0.05. **adjusted *P* < 0.01. ***adjusted *P* < 0.001. ****adjusted *P* < 0.0001. *n.s* not significant

The expression of genes between the groups is shown as scatter plots (Additional file 1). In the PM group, genes associated with skeletal or cardiac muscle (*Nppa*, *Nppb*, *Bmp10*, and *Mb*) were down regulated (Fig. 5A–gene cluster 5, 7, 9, and 11; and Additional file 1: Fig. S1A). The PM also down regulated *Bpifa1*, which plays a role in the innate immune responses of the upper airways. In the PM/OVA group, *Cxcl3,* which plays a role in inflammation and as a chemoattractant for neutrophils, *Cxcl5,* which recruits and activates leukocytes, and *Ccl8,* which displays chemotactic activity for monocytes, lymphocytes, basophils and eosinophils, were highly up regulated, and muscle related genes, *Nppa*, *Nppb*, *Bmp10*, and *Mb* were also down regulated compared with those of the control group (Fig. 5A–gene cluster 5, 6, 7, 10, and 11; and Additional file 1: Fig. S1B). Most of the highly variable genes in the PM group were also up or down regulated in the PM/OVA group (gene cluster 5, 7, and 11), and genes in cluster 11 were more prominently up regulated in the PM/OVA than those in the PM group. However, compared with the PM group, *Rnase2a,* which encodes eosinophil-derived neurotoxin, *Ccl7* and *Ccl24,* which attract monocytes and eosinophils, were highly up regulated. *Irf7,* which plays a role in the innate immune response against DNA and RNA viruses, *Ly6i,* which is associated with T cell physiology, oncogenesis, and immunological regulation, were down regulated in the PM/OVA group (Fig. 5A–gene cluster 2, 3, 6, and 10; and Additional file 1: Fig. S1C).

In the PM/OVA/CPX group, chemokine genes (*Cxcl3*, *Cxcl5*, *Cxcl10, Ccl2*, and *Ccl4*) down regulated in PM/OVA/CPX than in PM/OVA. However, inflammation and allergy associated genes (*Chil3* and *Chil4*), eosinophil-associated gene (*Ear7*), and B lymphocyte-associated gene (*Jchain*) were prominently up regulated in the PM/OVA/CPX group than in the PM/OVA group (Fig. 5A—gene cluster 8).

In the PM/OVA/Dex group, *Nppa, Chil4, Clca1, and Bpifa1* were highly up regulated. On the contrary, *Cxcl10, Ccl4, Ccl2, and Cxcl5* were down regulated compared with those of the PM/OVA group (Fig. 5A—gene cluster 1, 3, 5, 10, and 11; and Additional file 1: Fig. S1E). Especially, genes in cluster 1 were prominently up regulated than those in all other groups.

### Gene ontology analysis between groups

The analysis for Gene Ontology (GO)-enrichment in the biological process category is shown in the heatmap plot and upset plots (Fig. 5B; Additional file 1: Fig. S2). The result demonstrated that the enriched GOs in the PM and PM/OVA group were up regulated in the activation of immune systems, such as immune cells migration and chemotaxis, cytokine or chemokine-mediated inflammation, and antigen processing and presentation.

In both the PM/OVA/CPX and PM/OVA/Dex groups, granulocyte migration/chemotaxis, neutrophil migration/chemotaxis, acute-phase response, chemokine-medicated signaling pathway, and response to chemokine were significantly down regulated compared with that of the PM/OVA group. However, antigen processing and presentation (of peptide antigen), (positive) regulation of leukocyte-mediated immunity, leukocyte-mediated cytotoxicity, and regulation of innate immune response of the PM/OVA/CPX group were not significantly different from those of the PM/OVA group. On the contrary, these pathway activities of the PM/OVA/Dex group were significantly down regulated compared with those of the PM/OVA group. Additionally, chemokine-mediated signaling pathway, (cellular) response to chemokine, response to interferon-gamma, (regulation of) adaptive immune response, (regulation of) leukocyte mediated immunity, lymphocyte-mediated immunity, adaptive immune response based on somatic recombination of immune receptors built from immunoglobulin superfamily domains, and (defense) response to virus were more down regulated in the PM/OVA/CPX group than those in the PM/OVA group. Common up or down regulated pathways in the top 10 significant GOs between PM/OVA vs. control group, PM/OVA/CPX vs. PM/OVA, and PM/OVA/Dex vs. PM/OVA groups were neutrophil migration, granulocyte chemotaxis, myeloid leukocyte migration, and leukocyte chemotaxis (Fig. 5C).

Muscle related GOs, including striated or cardiac muscle, were down regulated in all experimental groups compared with those in the control group (Fig. 5B). Although significant down regulation was found in the PM/OVA/Dex group compared with that in the control group, the PM/OVA/Dex group tended to more upregulate muscle related GO activity than PM/OVA or PM/OVA/CPX group.

### Asthma and asthma-related pathway analysis with the KEGG database

**Fig. 6:**
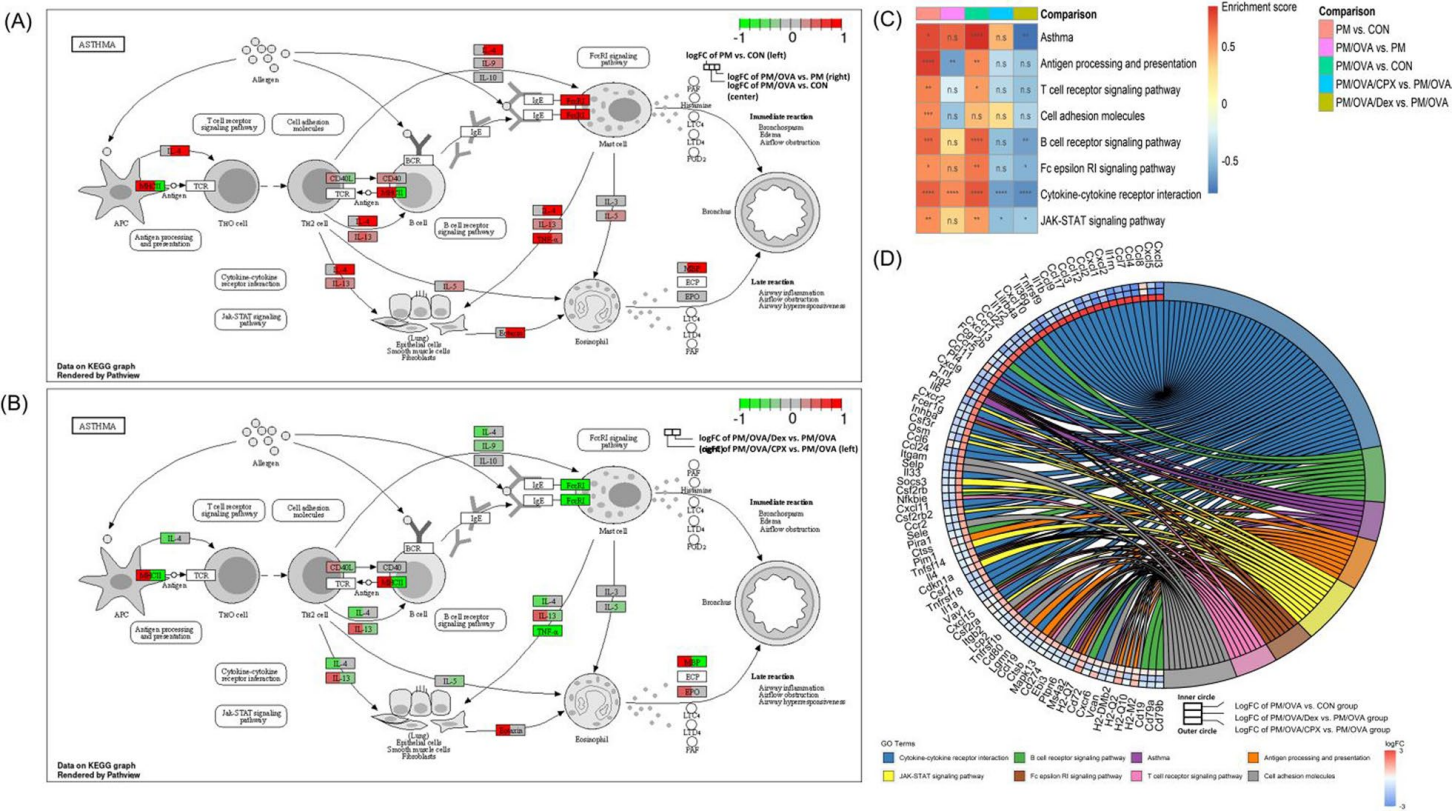
Asthma-related pathway activity and gene expression. Pathophysiology of asthma in exposure of PM, OVA, and PM/OVA with the KEGG database (**A**), effect of CPX and Dex on asthma pathway with the KEGG database (**B**), heatmap of asthma and asthma-related pathway enrichment score in each groups (**C**), chord diagram presenting differently expressed genes in asthma-related pathway of PM/OVA, PM/OVA/CPX, and PM/OVA/Dex (**D**). *adjusted *P* < 0.05. **adjusted *P* < 0.01. ***adjusted *P* < 0.001. ****adjusted *P* < 0.0001. *n.s* no significantly different

The single exposure of PM affected the immediate reaction of asthma by upregulation of the antigen processing and presentation via MCHII and the FcεRI in the mast cell (Fig. 6A). In the comparison between the PM/OVA and PM groups, OVA affected both the immediate and late reaction of asthma by upregulation of the interleukin-4 (IL-4), FcεRI, eotaxin, and MBP in asthma pathway, and cytokine-cytokine receptor interaction pathway (Fig. 6A, C, respectively). The concurrent exposure of PM and OVA significantly increased the entire asthma pathway and associated genes (Fig. 6A, D).

CPX decreased the expression of Il-4, Il-9, and FcεRI on immediate asthmatic reaction, and TNF-α on late asthmatic reaction. On the contrary, CPX increased the expression of MHCII, CD40L, Il-13, eotaxin (*Ccl11*), *Mbp, and Epo* on late reaction (Fig. 6B); therefore, CPX did not significantly decrease the entire asthma pathway (Fig. 6C). Dexamethasone decreased the expression of MHCII, CD40L, Il-13, Il-9, and FcεRI on immediate reaction, and TNF-α, Il-5, and MBP on late reaction (Fig. 6B). In addition, dexamethasone was significantly decreased in the entire asthma pathway, B cell receptor signaling pathway, and FcεRI signaling pathway, cytokine-cytokine receptor interaction, and JAK-STAT signaling pathway (Fig. 6C; Additional file 3). Notably, cytokine-cytokine receptor interaction and the JAK-STAT signaling pathway are commonly decreased pathways between PM/OVA/CPX and PM/OVA/Dex.

## Discussion

This study confirmed that CPX can improve PM_10_- and OVA-induced airway inflammation and fibrosis. Expression of chitinase-1 on airway epithelial cell and interstitial cells were suppressed by CPX. CPX also suppressed mRNA expression of chitinase-1 and level of chitinase-1 in lung homogenates. The suppression of chitinase-1 extensively altered mRNA expression associated with immune and inflammatory cascade. Specifically, CPX regulated asthma and asthma-related pathway including JACK-STAT signaling pathway. This ameliorates PM_10-_ and OVA-induced and aggravated airway inflammation and fibrosis as demonstrated by the changes in cytokine levels, pathologic findings, and airway hyper-responsiveness. We already know that PM_10_ can induce and aggravate respiratory diseases; however, there has been no therapeutic interventions in this case, until now. We believed that CPX can be a good candidate as a therapeutic option for PM_10_-associated respiratory disease.

We demonstrated a detailed pathway of how CPX can alter PM_10_- and OVA-induced airway inflammation and fibrosis using transcriptome. CPX extensively altered immune and inflammatory processes at the gene levels in the respiratory tract. Various genes were affected by CPX, including *Snord47*, *Snora68*, *Rnu11*, *Snora81 Cxcl3*, *Cxcl5*, *Cxcl10, Ccl2*, and *Ccl4*. CPX also decreased the expression of IL-4, IL-9, FcεRI, and TNF-α, which are associated with asthma-related pathway [22, 23]. In addition, CPX suppressed JAK-STAT signaling pathway, as demonstrated in the dexamethasone-treated group. The JAK-STAT signaling pathway is known to be associated with asthma inflammation, and JAK inhibitor is considered to have the potential to relieve asthma inflammation [24]. Extensive alterations of mRNA expression in lung tissue by CPX might lead to improved therapeutic effects on PM_10_- and OVA-induced airway inflammation and fibrosis.

Dexamethasone showed improvement of PM_10_-induced airway inflammation and fibrosis, similar to CPX. Dexamethasone, which is called a “cure-all,” has been widely used to treat various diseases, including allergic, inflammatory, and autoimmune diseases [25]. Therefore, we used dexamethasone as a positive control to treat PM_10_-induced airway diseases, and it also showed positive effects on it. However, dexamethasone cannot be used frequently and continuously since it can elicit various side effects, including hypertension, peptic ulcer, psychological disturbance, osteoporosis, susceptibility to infections, and adrenal insufficiency [26, 27]. Therefore, we believe dexamethasone cannot be easily prescribed in PM_10_-related airway disease. Contrary to dexamethasone, as a hormone which regulates overall homeostasis and immunity, CPX targets chitinase-1 specifically. We could not observe side effects of CPX, including weight decrease and mortality, in this study. We believe that CPX can be good candidate as a therapeutic option for PM_10_-associated respiratory diseases, as an alternative to dexamethasone.

We postulate that CPX can be used in other respiratory inflammatory and fibrotic diseases since CPX has a fundamental anti-bacterial effects [28]. Previous studies have also revealed that suppression of chitinase-1 can suppress AHR, inflammation, fibrosis, and improve airway remodeling [13, 15]. Lee et al. revealed that a CPX, kasugamycin, has strong antifibrotic effects to treat pulmonary fibrosis via the TGF-β signaling pathway [29]. Several studies have revealed the potential benefits of CPX in various respiratory diseases, including tuberculosis, sarcoidosis, chronic obstructive lung disease, and cystic fibrosis [30]. Although we demonstrated improvement of PM_10_-associated respiratory tract disease by CPX, it might have beneficial effects on other inflammatory and fibrotic disease. We think further studies can be conducted to extend the treatment of target diseases by CPX.

PM_10_ is a major air pollutant, and exposure to chronic PM_10_ can induce, facilitate, and aggravate airway inflammation and fibrosis [31, 32]. This also alters defense mechanisms and innate immunity in the lungs [33]. However, there are no studies on therapeutic options in PM_10_-associated respiratory disease. This study showed that CPX can be good therapeutic candidate for PM_10_-induced and aggravated airway inflammation and fibrosis. We also demonstrated the underlying pathway and mechanisms using mRNA sequencing. Considering the technical limitations of bulk RNA sequencing, additional experiments are needed to verify the results of this study and correct the unrecognized errors that may exist in the design of this study. Although further repeated studies and detailed studies on mechanisms are needed to confirm it, we believe that this study has made an important suggestion for future research directions. This study can also encourage exploration of further therapeutic agents for PM_10_-associated airway diseases.

To the best of our knowledge, this is the first study to suggest a significant role of a chitinase-1 inhibitor in PM_10_-induced airway inflammation. However, this study has several limitations. First, we only showed one timepoint experiment here. We previously published the results of 2 weeks of PM_10_ administration, and the results were similar to those of the 3 weeks of PM_10_ administration used in this study. Additionally, it usually takes at least 3 weeks to induce airway inflammation with OVA. Therefore, we chose 3 weeks administration of PM_10_ and OVA in this study, as a single timepoint experiment. Second, primary culture experiments using human airway epithelial cell from asthma patients will be needed to strengthen our hypothesis*.* Third, further detailed experiments are needed to reveal how chitinase-1 plays a role in PM_10_-induced lung inflammation. However, we attempted to indirectly assess the role of chitinase-1 by using the following methods: (1) observing the effects of chitinase-1 inhibitor and (2) analyzing asthma and asthma-related pathway using the KEGG database.

## Conclusion

We showed that chitinase-1 suppression by CPX can regulate PM_10_- and OVA-induced and aggravated airway inflammation and fibrosis via an asthma-related signaling pathway. This suggests that CPX can be good candidate for PM_10_-associated airway disease.

## Supplementary Information

**Additional file 1: Fig. S1. **Differential gene expression between groups. PM group vs. CON group (A), PM/OVA group vs. CON group (B), PM/OVA group vs. PM group (C), PM/OVA/CPX group vs. PM/OVA group (D), PM/OVA/DEXA group vs. PM/OVA group (E).

**Additional file 2: Fig. S2. **Upset plot of gene ontology analysis.

**Additional file 3: Fig. S3. **Asthma-related pathway analysis with the KEGG database. Cytokine-cytokine receptor interaction (PM vs. CON, PM/OVA vs. CON, and PM/OVA vs. PM) (A), Cytokine-cytokine receptor interaction (PM/OVA/CPX vs. PM/OVA and PM/OVA/Dex vs. PM/OVA) (B), Cell adhesion molecules (PM vs. CON, PM/OVA vs. CON, and PM/OVA vs. PM) (C), Cell adhesion molecules (PM/OVA/CPX vs. PM/OVA and PM/OVA/Dex vs. PM/OVA) (D), Antigen processing and presentation (PM vs. CON, PM/OVA vs. CON, and PM/OVA vs. PM) (E), Antigen processing and presentation (PM/OVA/CPX vs. PM/OVA and PM/OVA/Dex vs. PM/OVA) (F), JAK-STAT signaling pathway (PM vs. CON, PM/OVA vs. CON, and PM/OVA vs. PM) (G), JAK-STAT signaling pathway (PM/OVA/CPX vs. PM/OVA and PM/OVA/Dex vs. PM/OVA) (H), T cell receptor signaling pathway (PM vs. CON, PM/OVA vs. CON, and PM/OVA vs. PM) (I), T cell receptor signaling pathway (PM/OVA/CPX vs. PM/OVA and PM/OVA/Dex vs. PM/OVA) (J), B cell receptor signaling pathway (PM vs. CON, PM/OVA vs. CON, and PM/OVA vs. PM) (K), B cell receptor signaling pathway (PM/OVA/CPX vs. PM/OVA and PM/OVA/Dex vs. PM/OVA) (L), FcεRI signaling pathway (PM vs. CON, PM/OVA vs. CON, and PM/OVA vs. PM) (M), FcεRI signaling pathway (PM/OVA/CPX vs. PM/OVA and PM/OVA/Dex vs. PM/OVA) (N).

## Data Availability

The datasets used and analyzed during the current study are available from the corresponding author on reasonable request.
