# Supplementary figures and images for: Particulate matter10-induced airway inflammation and fibrosis can be regulated by chitinase-1 suppression
Source: Respir Res. 2023 Mar 18;24:85. doi: 10.1186/s12931-023-02392-8 (PMC10024831; doi:10.1186/s12931-023-02392-8)

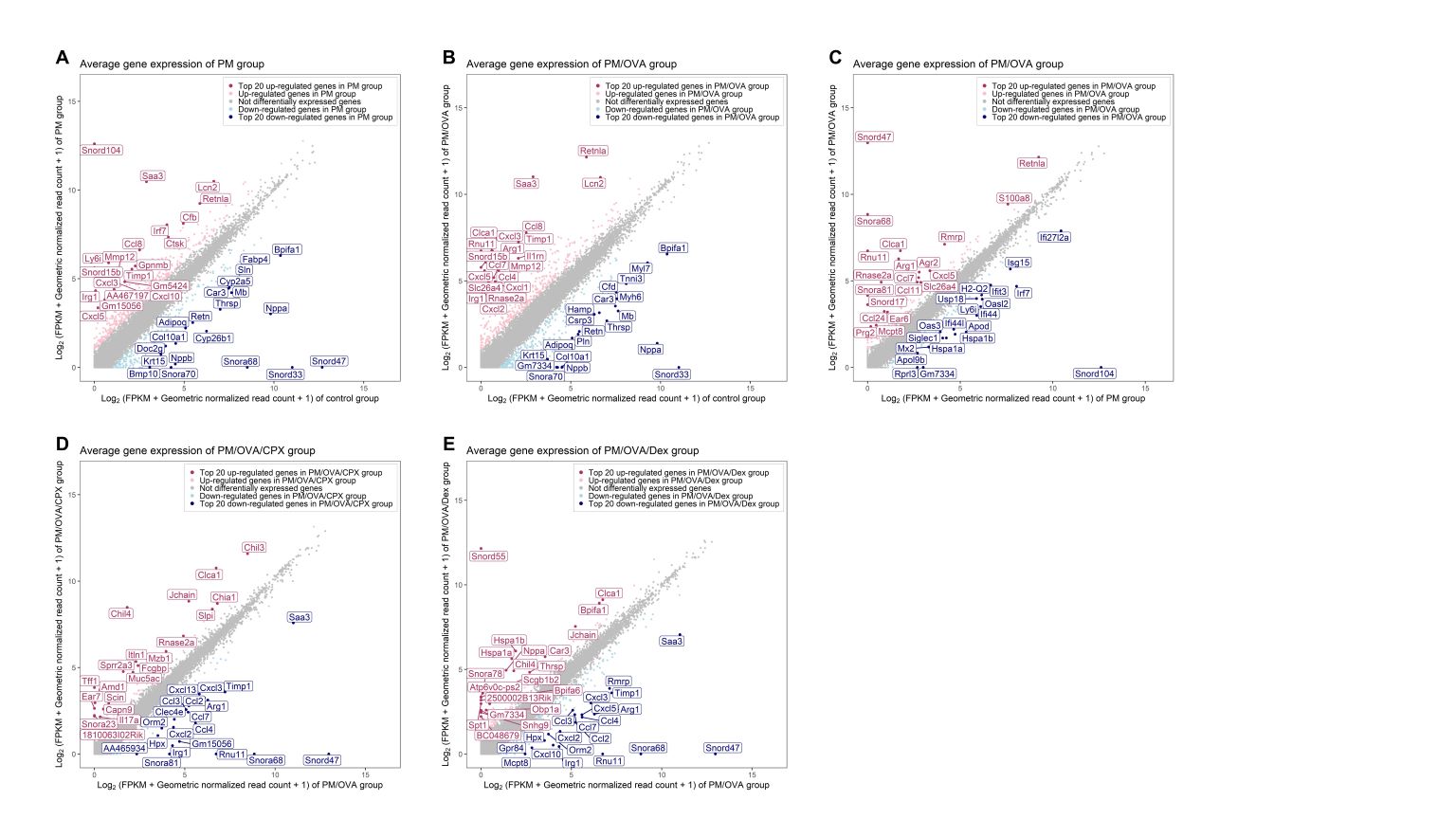

Supplement: Supplementary file 1 — Additional file 1: Fig. S1. Differential gene expression between groups. PM group vs. CON group (A), PM/OVA group vs. CON group (B), PM/OVA group vs. PM group (C), PM/OVA/CPX group vs. PM/OVA group (D), PM/OVA/DEXA group vs. PM/OVA group (E). [file 12931_2023_2392_MOESM1_ESM.jpg]

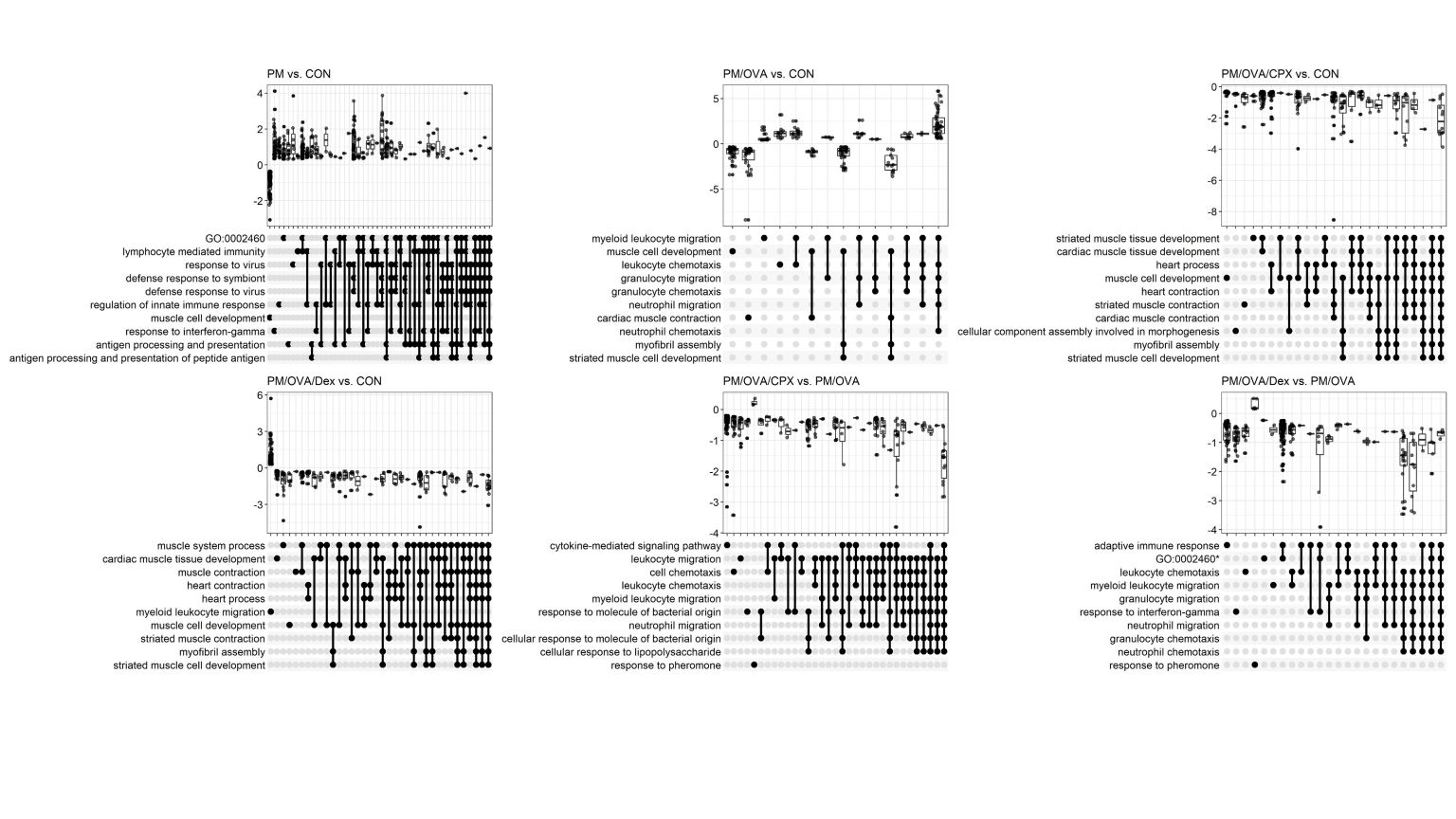

Supplement: Supplementary file 2 — Additional file 2: Fig. S2. Upset plot of gene ontology analysis. [file 12931_2023_2392_MOESM2_ESM.jpg]

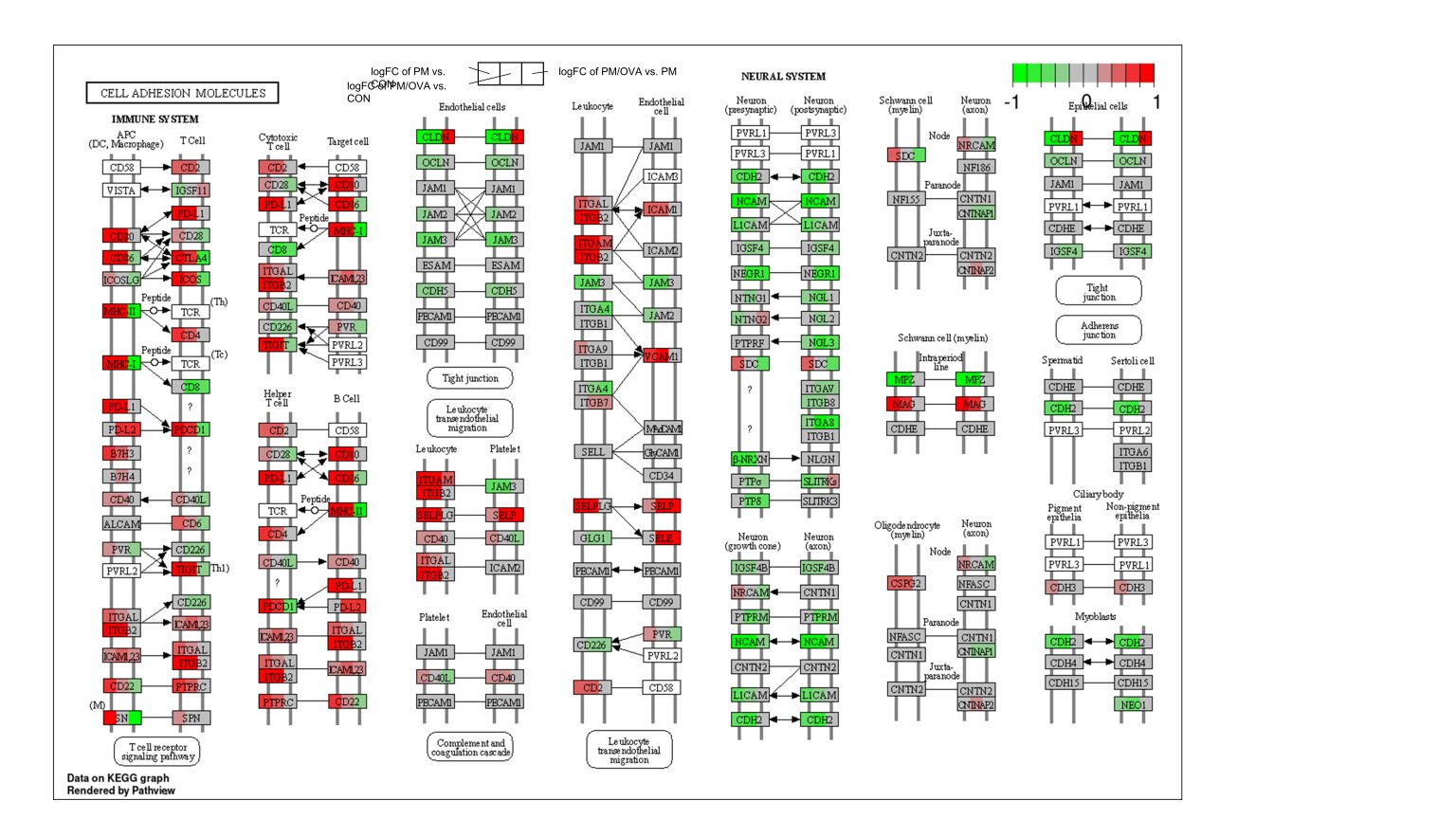

Supplement: Supplementary file 3 — Additional file 3: Fig. S3. Asthma-related pathway analysis with the KEGG database. Cytokine-cytokine receptor interaction (PM vs. CON, PM/OVA vs. CON, and PM/OVA vs. PM) (A), Cytokine-cytokine receptor interaction (PM/OVA/CPX vs. PM/OVA and PM/OVA/Dex vs. PM/OVA) (B), Cell adhesion molecules (PM vs. CON, PM/OVA vs. CON, and PM/OVA vs. PM) (C), Cell adhesion molecules (PM/OVA/CPX vs. PM/OVA and PM/OVA/Dex vs. PM/OVA) (D), Antigen processing and presentation (PM vs. CON, PM/OVA vs. CON, and PM/OVA vs. PM) (E), Antigen processing and presentation (PM/OVA/CPX vs. PM/OVA and PM/OVA/Dex vs. PM/OVA) (F), JAK-STAT signaling pathway (PM vs. CON, PM/OVA vs. CON, and PM/OVA vs. PM) (G), JAK-STAT signaling pathway (PM/OVA/CPX vs. PM/OVA and PM/OVA/Dex vs. PM/OVA) (H), T cell receptor signaling pathway (PM vs. CON, PM/OVA vs. CON, and PM/OVA vs. PM) (I), T cell receptor signaling pathway (PM/OVA/CPX vs. PM/OVA and PM/OVA/Dex vs. PM/OVA) (J), B cell receptor signaling pathway (PM vs. CON, PM/OVA vs. CON, and PM/OVA vs. PM) (K), B cell receptor signaling pathway (PM/OVA/CPX vs. PM/OVA and PM/OVA/Dex vs. PM/OVA) (L), FcεRI signaling pathway (PM vs. CON, PM/OVA vs. CON, and PM/OVA vs. PM) (M), FcεRI signaling pathway (PM/OVA/CPX vs. PM/OVA and PM/OVA/Dex vs. PM/OVA) (N). [file 12931_2023_2392_MOESM3_ESM.jpg]
